# Biodegradable Polymersomes for the Delivery of Gemcitabine to Panc-1 Cells

**DOI:** 10.1155/2013/932797

**Published:** 2013-06-23

**Authors:** Nimil Sood, Walter T. Jenkins, Xiang-Yang Yang, Nikesh N. Shah, Joshua S. Katz, Cameron J. Koch, Paul R. Frail, Michael J. Therien, Daniel A. Hammer, Sydney M. Evans

**Affiliations:** ^1^Department of Chemical and Biomolecular Engineering, University of Pennsylvania, Philadelphia, PA, USA; ^2^Department of Radiation Oncology, University of Pennsylvania, Perelman School of Medicine, Philadelphia, PA, USA; ^3^Department of Neuroscience, University of Miami, Coral Gables, FL, USA; ^4^Department of Bioengineering, University of Pennsylvania, Philadelphia, PA, USA; ^5^Department of Chemistry, University of Pennsylvania, Philadelphia, PA, USA; ^6^Department of Chemistry, Duke University, Durham, NC, USA

## Abstract

Traditional anticancer chemotherapy often displays toxic side effects, poor bioavailability, and a low therapeutic index. Targeting and controlled release of a chemotherapeutic agent can increase drug bioavailability, mitigate undesirable side effects, and increase the therapeutic index. Here we report a polymersome-based system to deliver gemcitabine to Panc-1 cells *in vitro*. The polymersomes were self-assembled from a biocompatible and completely biodegradable polymer, poly(ethylene oxide)-poly(caprolactone), PEO-PCL. We showed that we can encapsulate gemcitabine within stable 200 nm vesicles with a 10% loading efficiency. These vesicles displayed a controlled release of gemcitabine with 60% release after 2 days at physiological pH. Upon treatment of Panc-1 cells *in vitro*, vesicles were internalized as verified with fluorescently labeled polymersomes. Clonogenic assays to determine cell survival were performed by treating Panc-1 cells with varying concentrations of unencapsulated gemcitabine (FreeGem) and polymersome-encapsulated gemcitabine (PolyGem) for 48 hours. 1 *μ*M PolyGem was equivalent in tumor cell toxicity to 1 *μ*M FreeGem, with a one log cell kill observed. These studies suggest that further investigation on polymersome-based drug formulations is warranted for chemotherapy of pancreatic cancer.

## 1. Introduction

Pancreatic adenocarcinoma is the fourth highest cause of cancer death with a 5-year survival rate of less than 6% [1]. Despite the use of surgery, radiation, and/or chemotherapy [2], local recurrence and metastasis invariably occur. The causes of resistance of pancreatic tumors are not completely understood. The inability to deliver adequate adjuvant therapy due to local normal tissue toxicity, limitations caused by tumor microenvironment (hypoxia, pH), and active drug export out of tumor cells likely cause this resistance [3, 4]. Modifications to the delivery of chemotherapeutics that improve the therapeutic ratio (TR) are highly desirable in order to allow higher drug delivery while minimizing toxicity to normal tissues.

Gemcitabine is a commonly used water soluble anticancer agent that acts as an antimetabolite; it is considered an efficacious addition to radiation therapy in pancreatic cancer [5]. Gemcitabine is an S-phase deoxycytidine analog (2′,2′-difluorodeoxycytidine). Its mechanism of action involves competitive incorporation into DNA, masked termination (causing termination of DNA synthesis without being excised out of the strand), and self-potentiation (promoting its own activity by inhibiting regulatory enzymes involved in DNA synthesis). Like most chemotherapeutics, its use has significant limitations. Gemcitabine is rapidly metabolized in the blood stream with a short plasma half-life (for short infusions, 32–94 min) and has substantial side effects that limit the dose that can be given, especially when combined with radiation therapy [6]. In a phase 1 study, concurrent application of gemcitabine and radiation caused nausea, vomiting, dehydration, and gastric ulceration resulting in a 44% hospital admission rate [7]. These side effects are much greater for concurrent therapy than for just radiation, which has been linked primarily to nausea. Encapsulation of gemcitabine in a carrier vehicle has the potential to reduce dose-limiting side effects while improving the drug delivery to the tumor. The latter includes increased circulation time and preferential accumulation in tumor due to the enhanced permeability and retention (EPR) effect [8].

 Encapsulation of gemcitabine to address the challenges of rapid blood metabolism and low therapeutic ratio has been previously investigated. In one study, gemcitabine loaded in sonochemically prepared bovine serum albumen (BSA) microspheres was evaluated for cell killing in renal cancer *in vitro* [9]. These microspheres exhibited poor dynamics of release and were unable to take advantage of the EPR effect observed in solid tumors due to their large size (~1 *μ*m diameter), an effect which requires 150–300 nm particles in diameter [8]. In another study, albumin nanospheres were loaded with gemcitabine [10]; this delivery system also had poor release kinetics with 100% of the drug being released in 24 hours. In a third study, encapsulated gemcitabine within poly(ethylene glycol)-poly(DL-lactic acid) (PEG-PDLLA) nano vesicles showed toxicity against SW1990 pancreatic cells [11]. The vesicle morphology and size of these vesicles were very variable. 

Polymer vesicles, or polymersomes, are self-assemblies of amphiphilic block copolymers that can encapsulate both hydrophobic and hydrophilic compounds [12, 13]. Their highly tunable chemistry allows for diverse functionalities and applications [14]. Polymersomes possess superior biomaterial properties compared to their lipid counterparts (liposomes) including greater stability, storage capacity, release characteristics, and plasma circulation times [15–18]. The hydrophilic block is often composed of poly(ethylene oxide) (PEO) head groups, which helps reduce non-specific interactions with blood proteins due to their hydrophilicity and steric hindrance effects. This greatly reduces opsonization of nanoparticles and increases their plasma circulation time. Several biodegradable hydrophobic blocks can be utilized for drug delivery including polycaprolactone (PCL) and polylactide (PLA) for polymersomes and polylactic-co-glycolic acid (PLGA) for nanoparticles [14, 19–21]. PCL has several advantages over the other polymers including high permeability to small molecules, maintenance of neutral pH after degradation, ease of blending with other polymer blocks, and long-term and tunable erosion kinetics [22].

Recognizing the potential of PEO-PCL polymersomes for use in cancer treatment, this paper describes the novel use of PEO-PCL nanopolymersomes for gemcitabine encapsulation and *in vitro* delivery to Panc-1 cells. We investigated the polymersome release kinetics of gemcitabine, vesicle internalization by Panc-1 cells, and cell toxicity of PolyGems compared to standard gemcitabine (FreeGem). Polymersomes were internalized by Panc-1 cells and had equivalent cell toxicity at the same total dose when loaded with gemcitabine. These results suggest that PolyGems have the potential to be an attractive route to improve gemcitabine delivery *in vivo*.

## 2. Materials and Methods

### 2.1. Materials

Gemcitabine (Gemzar) was obtained from Eli Lilly and Company (Indianapolis, IN). Panc-1 cells were obtained from the ATCC. DMEM/F12 Ham's (50/50) without phenol red was purchased from Zen-Bio (Research Park, NC). The meso-to-meso ethyne-bridged (porphinato) zinc (II) trimer (PZn_3_) with a 9-methoxy-1,4,7-trioxanonyl substituent on one aryl group and a more hydrophobic 3,3-dimethyl-1-butyloxy substituent on the other was synthesized as previously described [23]. Glacial acetic acid, methylene chloride, methylene blue for colony staining, phosphate buffered saline (PBS), sodium acetate trihydrate, and sodium chloride were purchased from Fisher Chemicals (Pittsburgh, PA). Polycarbonate extrusion membranes (13 mm) were purchased from Whatman (Piscataway, NJ). Centrifugal filter units were purchased from Millipore (Billerica, MA). Dialysis cassettes were purchased from Spectrum Laboratories (Rancho Dominguez, CA).

### 2.2. PEO-PCL Preparation

PEO-*b*-PCL with 45 and 105 monomer repeat units per block, respectively, (MW = 14,000 g mol^−1^) was synthesized prior to this work [14]. The block copolymer was generated via ring-opening polymerization of cyclic *ε*-caprolactone. Briefly, monomethoxypoly(ethylene oxide) (2k) was filled in a flame-dried flask under argon. Caprolactone monomer was injected into the flask via syringe, and two drops of stannous (II) octoate was added to the reaction mixture. The reaction occurred at 130°C for 24 hours. The copolymer was isolated by dissolving the product in methylene chloride and precipitating in excess methanol/hexane at 4°C. The resulting powder was dried further. The block copolymer was purified via gel permeation chromatography, and the molecular weight was determined by ^1^H NMR.

### 2.3. Vesicle Preparation

Polymersomes were synthesized by the thin film hydration method as described elsewhere [14]. Briefly, 200 *μ*L of a 100 mg/mL PEO-PCL solution in methylene chloride were deposited on a roughened teflon strip and allowed to dry overnight under vacuum. A 2.21 mg/mL (corresponding to 5 : 1 molar ratio of drug : polymer) solution lyof gemcitabine in 0.9% saline (~290 mOsm) was added to the film in a 20 mL vial in order to hydrate the polymer. Polymersomes were formed by one hour of sonication at 60°C and 5 freeze/thaw cycles using liquid nitrogen. A narrow size distribution of polymersomes was obtained by successive extrusion through 400 nm, 200 nm, and 100 nm membranes using a thermobarrel extruder (Lipex Biomembranes, Vancouver, Canada) operating at 65°C. Size was verified using a Nano Zs Zetasizer (Malvern Instruments, Southborough, MA). 0.9% saline without drug was used as the hydration solution for control studies. For cellular uptake studies, polymersomes were loaded with the porphyrin-based near-IR fluorophore, PZn_3_ (*λ*_ex_ = 785 nm, *λ*_em_ = 800 nm), at a molar ratio of 40 : 1 polymer to PZn_3_ by cocasting the PZn_3_ with the polymer film. Before cell culture studies, vesicles were sterilized for 30 minutes via germicidal UV irradiation.

### 2.4. Cryo-TEM

Cryogenic transmission electron microscopy was performed at the University of Pennsylvania in the Penn Regional Nanotechnology Facility (Philadelphia, PA). Lacey formvar/carbon grids (Ted Pella) were rinsed with chloroform to remove the formvar template and subsequently carbon coated with a Quorum Q150T ES carbon coater (Quorum Technologies, UK). Grids were cleaned with hydrogen/oxygen plasma for 15 seconds using the Solarus Advanced Plasma System 950 (Gatan, Pleasanton, CA). Polymersome sample (2 *μ*L) was deposited on lacey formvar/carbon mesh grid (Ted Pella) and inserted into a cryoplunger (Gatan Cp3, Gatan). The sample was blotted by hand and plunged into liquid ethane. Samples were transferred to a cryoholder (Gatan CT3500TR, Gatan), and the cryoholder was immediately inserted into a JEOL 2010 TEM (JEOL) operating at 200 kV. Micrographs were imaged with an Orius SC200 digital camera.

### 2.5. Vesicle Release Kinetics

Nanovesicles were prepared as described above. After extrusion, vesicles were concentrated to 0.5 mL volume using 3 kDa centrifugal filters made from regenerated cellulose. Additional removal of unencapsulated drug was performed via dialysis against a pH 5.0 sodium acetate/sodium chloride buffer (acidified with glacial acetic acid) or pH 7.4 PBS buffer. Immediately following dialysis, 250 *μ*L sample aliquots were placed in microdialysis tubes and stored in 22 mL of pH 5.0 or 7.4 buffer in a 37°C oven. At predefined time points, aliquots were taken from the buffer solution and read on a UV/Vis spectrophotometer at 270 nm (*ε* = 9.86 × 10^−3 ^cm^−1^ *μ*M^−1^). Aliquots were returned to the buffer to maintain a constant volume. 100% release was determined by addition of 100 *μ*L of a 1% Triton-X solution to the PolyGem vials after one week, and the gemcitabine absorbance after overnight storage at 37°C was measured.

### 2.6. Cell Culture

At the time of receipt from the ATCC, cells were placed in cryovials and stored in liquid nitrogen for future use. Cells for study were defrosted using standard procedures. New cell stock was defrosted at 6-month intervals. Panc-1 cells were cultured in DMEM/F12 Ham's (50/50) without phenol red and with 12% fetal calf serum and 1% Pen/Strep. Cells were maintained in T75 plastic culture flasks at 37°C in a humidified atmosphere containing 5% CO_2_ in air. Flasks were subcultured when they were 75–90% confluent. Five minute exposure to 0.05% trypsin-EDTA was used to release attached cells from the tissue culture surface.

### 2.7. *In Vitro* Cellular Uptake of Vesicles

Fluorescent polymersomes were synthesized as previously described. The concentration of PZn_3_ was determined by Beer's law using the established extinction coefficient *ε*(795 nm) = 1.25 cm^−1^ *μ*M^−1^ [24]. Panc-1 cells were plated in triplicate at 5,000 cells per well in 96 well (black frame, clear well) cell culture plates (Isoplate-96 TC, Perkin Elmer). Cells were allowed to adhere overnight. The following day, varying concentrations of fluorescent polymersomes were added to wells and were incubated for 12, 24, and 48 hours. At each time point, one plate was removed from incubation washed three times with media to remove all polymersomes that were not internalized. Vesicles were illuminated on a LICOR Odyssey (*λ*_ex_ = 488 nm, *λ*_em_ = 810 nm). A calibration curve was generated to relate the fluorescence signal from wells to the concentration of PZn_3_ to determine cellular uptake. Confocal laser scanning microscopy (CLSM) was used to visualize vesicle internalization in Panc-1 cells incubated with fluorescent polymersomes for 12 hours. An Olympus FluoView FV1000 confocal microscope (Center Valley, PA) with a PLFLN 40x oil objective lens was used to obtain *z*-stacks of cells with a scan speed of 8.0 *μ*s pixel^−1^ and step size of 1 *μ*m. 

### 2.8. *In Vitro* Toxicity

Panc-1 cells were plated onto 60 mm tissue culture dishes. After 48 hours, the plates were examined under the microscope for evidence of cell growth. Only plates containing of 50–90% confluent cells were used for study. Following the removal of spent media, the plates were rinsed with 2 mL of fresh media. The rinsed media was then replaced with 2 mL of treatment solution (PolyGem, FreeGem, or blank polymersomes). Dishes were incubated for an additional 48 hours. At the time of assay, cells were removed from the plate by incubating 2 mL of 0.05% trypsin-EDTA with the cells for 5 minutes at 37°C. Trypsin was inactivated using fresh media with serum, and the total cell number was determined using a Coulter counter. A clonogenic assay was performed using standard techniques [25]. Plates were incubated for two weeks, with the goal of obtaining 25–250 colonies per plate. The surviving fraction was determined by normalizing the colony count of the treated condition by the initial cell number plated. The concentration of gemcitabine as reported for PolyGem represents the total encapsulated concentration. We corrected for the drug release relative to time, which was 60% release at 48 hours. The survival graphs were presented in absolute fractions with the maximal plating efficiency of untreated Panc-1 cells being 0.60. All comparisons were made to this value. Qualitative morphology of treated cells was observed using a Zeiss inverted microscope. Panc-1 cells were treated with media, 5 *μ*M FreeGem, 5 *μ*M PolyGem, or 2.5 *μ*M blank polymersomes.

### 2.9. Statistical Analysis

All experiments were performed in triplicate, except where noted. *In vitro* toxicity studies were performed in duplicate with varying amounts of initial cell plating to ensure the resulting colonies were within a countable range (50–200 colonies). Data were reported as mean ± standard deviation and analyzed by single-factor ANOVA, setting the level of significance at *P* < 0.05.

## 3. Results

### 3.1. Vesicle Preparation and Characterization

Nanovesicles were prepared by thin-film hydration. This method is particularly attractive for making polymersomes for biological applications as it does not require the use of potentially toxic organic solvents during the hydration step (as is employed in the solvent injection method) [26].

**Figure 1:**
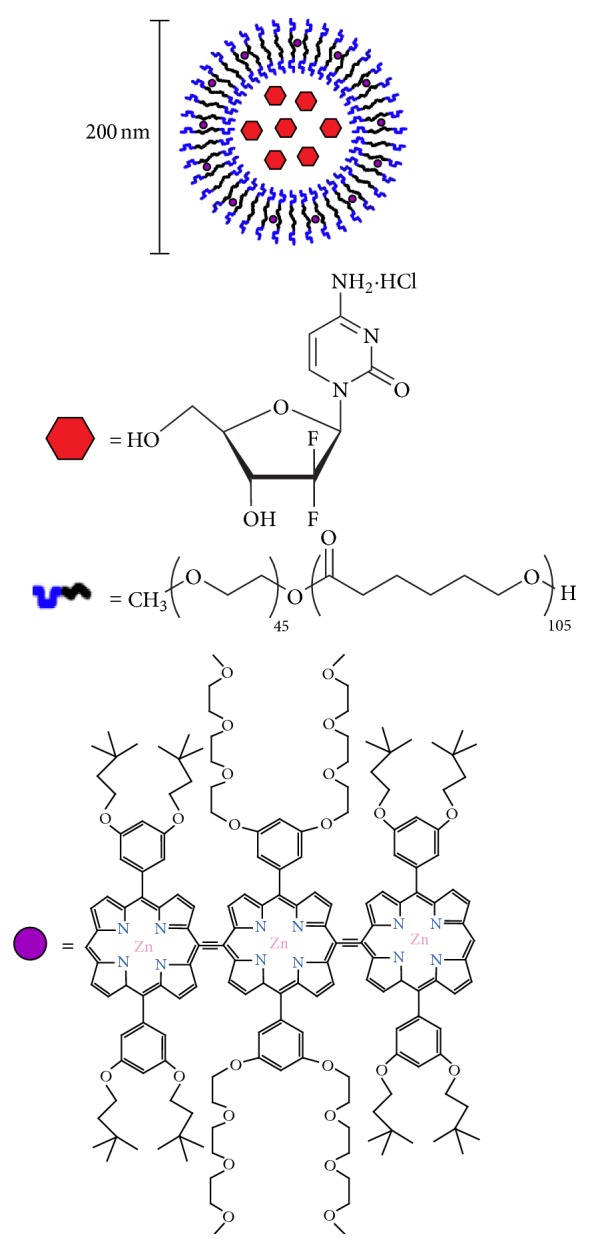
Schematic representation of PolyGem. In aqueous solution, poly(ethylene oxide)-b-poly(caprolactone) (PEO-PCL) self-assemble into spherical polymer vesicles (polymersomes), with the hydrophobic PCL chains orienting end to end to form the bilayer. The figure represents a uniaxial cross section of the polymersome, with gemcitabine (⎔) encapsulated in the aqueous lumen. Vesicles can also be made to include PZn_3_  (◯) in the hydrophobic membrane.

Figure 1 shows a schematic of our novel carrier. The size distribution of vesicles was measured using dynamic light scattering (DLS) and is shown in Table 1. Vesicles were serially extruded through 400, 200, 100, 100, and 200 nm polycarbonate membranes to reduce the sample polydispersity. The average hydrodynamic diameter of PolyGems was 180 nm. The hydrodynamic diameter of blank polymersomes (181 nm) and PZn_3_-polymersomes (180 nm) did not statistically vary from PolyGems (*P* > 0.05). Vesicles were stored at 4°C and used in the studies within one week of preparation. We evaluated the stability of gemcitabine after sonication and freeze/thaw cycles by checking the absorbance of the drug before and after processing. There was no difference in the absorbance spectrum, indicating that the drug retained its structure.

### 3.2. Vesicle Drug Release

**Figure 2:**
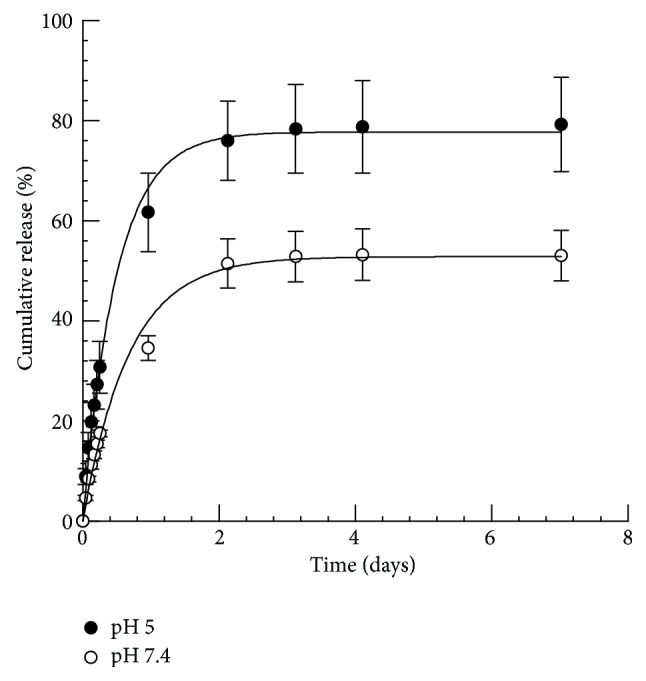
Cumulative *in situ* release of gemcitabine from PolyGem at pH 5.0 and 7.4 and 37°C, as measured via UV/Vis spectroscopy for 7 days. *n* = 3 for each data point and error bars represent standard deviation.

 We explored the release profile of gemcitabine from polymersomes at 37°C at pH 5.0 and 7.4. The results are shown in Figure 2. Approximately 20% of the gemcitabine is released from the PolyGems within the first few hours at both pHs. The concentration of gemcitabine released begins to level off at approximately day two, indicating stable and controlled release after an initial period up to 48 hours. More gemcitabine is released per unit time at pH 5.0 than at pH 7.4.

The release curves are well fit by a function of the form *c*/*c*_0_ = *α*(1 − *e* ^−*t*/*τ*^), where *α* is a lumped constant and *τ* is the characteristic time constant. This functional form can be derived from a 1D analysis of drug diffusion across a semipermeable membrane. Fitting this equation to the release curves in Figure 2 reveals that *τ*_pH5_ is 12 hours and *τ*_pH7_ is 16 hours (lower values of *τ* indicate more rapid release).

### 3.3. Cryo-TEM of Vesicles

**Figure 3:**
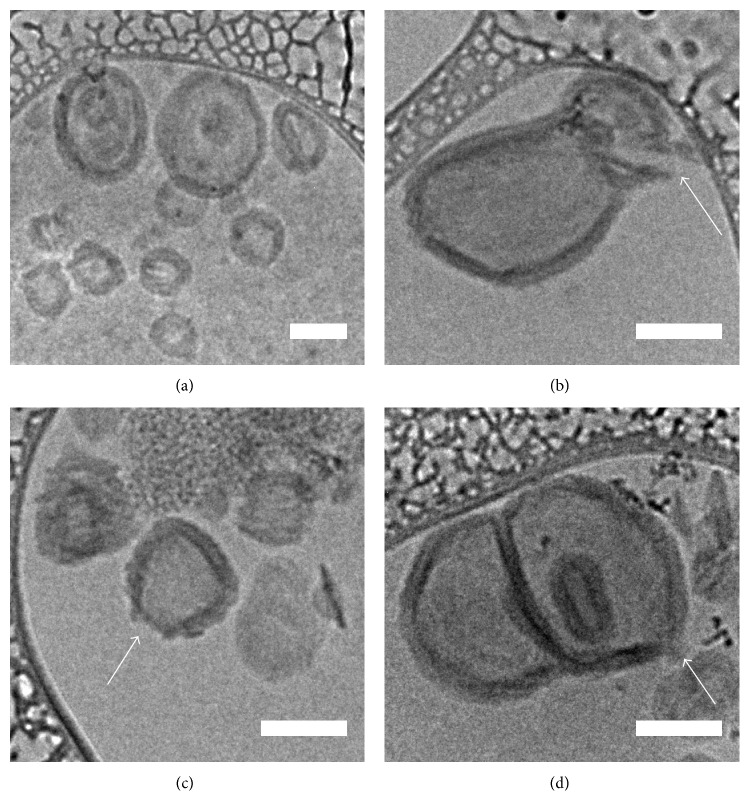
Cryo-TEM micrographs of PEO-PCL vesicles incubated for 12 hours. (a) pH 7.4. (b)–(d) pH 5.0. Arrows indicate areas of membrane degradation. Scale bar = 100 nm.

 In order to understand the difference in observed release rate between vesicles at pH 5.0 and 7.4, we observed the structure of nanovesicles using cryo-TEM at the incubation different conditions.  Figure 3 provides representative images of vesicles that have been incubated at either pH 7.4 (Figure 3(a)) or pH 5.0 (Figures 3(b)–3(d)) for 12 hours. The vesicles in Figure 3(a) have an even membrane thickness that is an indicative of an intact membrane. The vesicles in Figures 3(b)–3(d) have compromised membranes as shown by the arrows. In Figure 3(b), the membrane of one vesicle has completely disintegrated and has formed a pore.  Figure 3(c) shows a vesicle with a thinning portion of the membrane. Finally, in Figure 3(d) we see a membrane pore starting to form.

### 3.4. *In Vitro* Cellular Uptake

**Figure 4:**
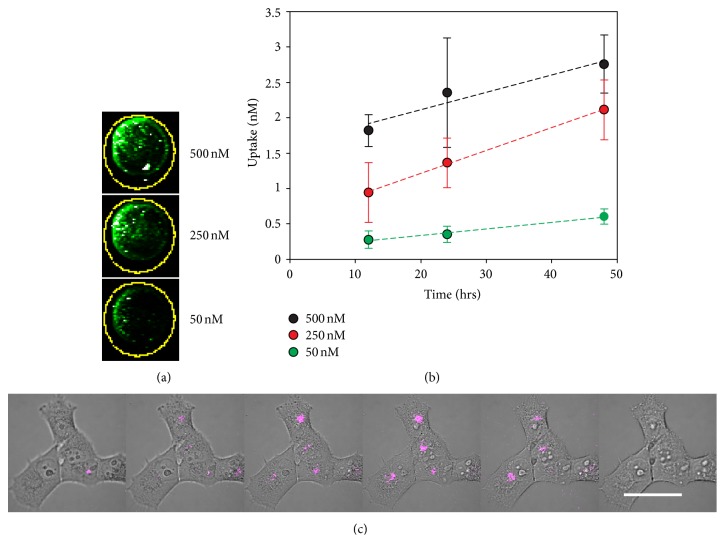
PolyGem internalization by Panc-1 cells. (a) Fluorescence intensity of PZn_3_-polymersomes internalized by cells in well plates corresponding to 48 hour time point. (b) Concentration of PZn_3_ uptake as a function of solution PZn_3_ concentration (*n* = 3). Error bars indicate standard deviation. (c) CLSM *z*-stack images of Panc-1 cells incubated with PZn_3_-polymersomes for 12 hours. Z-slices (Δ*z* = 3 *μ*m) are presented from left to right. Scale bar = 50 *μ*m.

We visualized polymersome internalization by a cellular uptake study of blank polymersomes loaded with a hydrophobic porphyrin-based NIR fluorophore, PZn_3_, in the vesicle membrane. In our study, Panc-1 cells were incubated with 50, 250, or 500 nM of PZn_3_-loaded polymersomes in 96-well plates for 12, 24, or 48-hours. A calibration was performed to relate the integrated intensity in wells to PZn_3_ concentration.  Figure 4 shows the concentration of internalized PZn_3_ as a function of incubation time. Vesicle uptake increased with an increase in the concentration of PZn_3_ as well as incubation time. In order to confirm that the internalization from the uptake study was not surface association, Panc-1 cells were incubated with 500 nM PZn_3_ for 12 hours and imaged on a confocal microscope with 1 *μ*m *z*-slices.  Figure 4(c) shows *z*-slices starting from the top of the cell and moving to the bottom. Vesicles are only observed internally and not on the cell surface.

### 3.5. *In Vitro* Cell Toxicity

**Figure 5:**
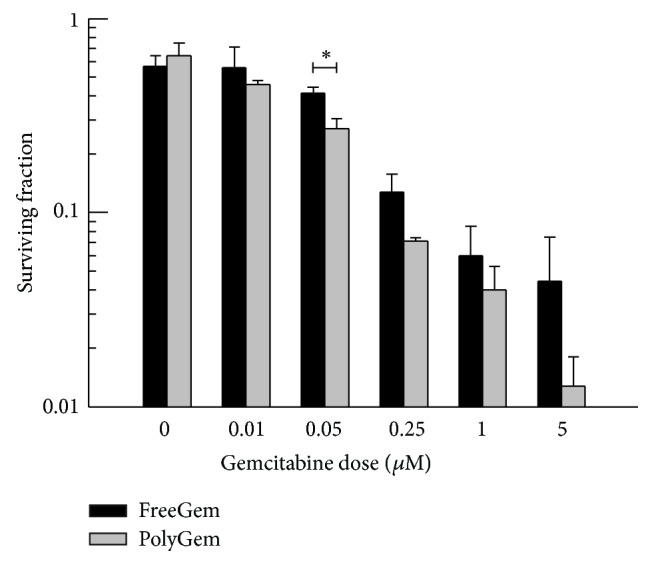
Panc-1 viability after 48 hour treatment with varying concentrations of FreeGem or PolyGem, as measured by a clonogenic assay. Error bars indicate standard deviation (*n* = 3). ∗*P* < 0.05.

 Panc-1 cell survival was determined by a clonogenic assay following 48-hour FreeGem or PolyGem exposure using varying drug concentrations (Figure 5). Panc-1 survival is concentration dependent with an observed increase in cell kill as gemcitabine concentration was increased irrespective of the formulation used. A one log cell kill is observed at approximately 1 *μ*M gemcitabine, irrespective of the formulation. The only drug concentration where there was a significant difference in survival between FreeGem and PolyGems was at 0.05 *μ*M gemcitabine (*P* = 0.048) where the PolyGem were more effective than the FreeGem. There was no significant cell kill using either blank polymersomes (0 *μ*M PolyGem) or media-only treatment (0 *μ*M FreeGem) as seen in Figure 5.

**Figure 6:**
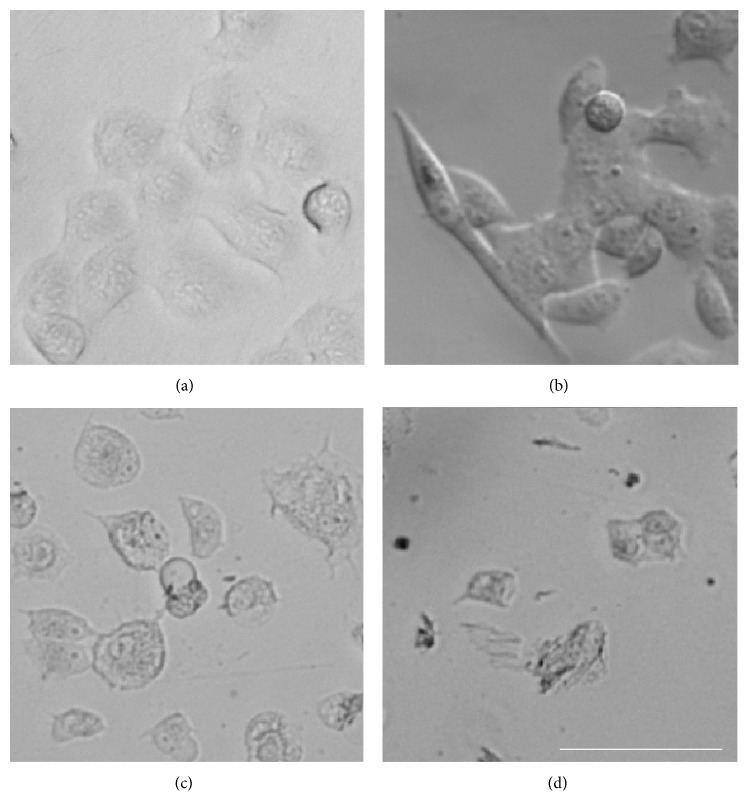
Cell phenotype as visualized by DIC. (a) Media control, (b) 2.5 *μ*M empty polymersomes, (c) 5 *μ*M FreeGem, and (d) 5 *μ*M PolyGem. Scale bar = 100 *μ*m.

To qualitatively assess cell toxicity, Panc-1 cells were imaged using differential interference contrast microscopy (DIC) following 48 hour exposure to media, 5 *μ*M FreeGem, 5 *μ*M PolyGem, or 2.5 *μ*M blank polymersomes. These doses of PolyGem, FreeGem, and blank polymersomes were chosen to reflect the maximum concentration of gemcitabine delivered to Panc-1 cells in the clonogenic assay and to equate the concentration of PEO-PCL across the formulations (2 : 1 ratio of drug to polymer). Figures 6(a) and 6(b) demonstrate that application either of media or blank polymersomes, respectively, has no effect on cell viability since the cells are morphologically normal. Both 5 *μ*M FreeGem and 5*μ*M PolyGem treatments (Figures 6(c) and 6(d), resp.) resulted in significant cell killing as observed by the presence of morphologically abnormal cells and cellular debris.

## 4. Discussion

 Despite the advancement of chemoradiation therapy for the treatment of pancreatic cancer, the 5-year survival rate continues to be among the lowest of solid tumors. Any improvement in the delivery of chemotherapeutics to pancreatic cancer should mitigate side-effects associated with treatment and improve the survival outlook for patients. In the studies reported herein, we created a prototype vehicle which could ultimately be capable of delivering gemcitabine, a potent pancreatic cancer drug, to pancreatic cancer. The major aim of this work was to perform a comparative *in vitro* study of the cell-killing efficacy of polymersome-encapsulated gemcitabine (PolyGem) and unencapsulated gemcitabine (FreeGem) on Panc-1 cells.

 Bioresorbable polymersomes were synthesized from two FDA-approved polymers, poly(ethylene oxide) (PEO) and poly(caprolactone) (PCL). The polymers self-assembled in a fashion such that the hydrophobic PCL block was embedded in the polymersome membrane and the PEO block was exposed on the outer vesicle surface and inner corona (Figure 1). The internal hydrophilic lumen provided an aqueous environment for gemcitabine encapsulation, while hydrophobic porphyrin molecules were located within the membrane of the vesicles. Encapsulation of gemcitabine or porphyrin into the polymersomes did not alter the hydrodynamic diameter as compared to blank polymersomes (Table 1). 

To improve the tumor-killing effect of drug delivery vesicles *in vivo*, maximal drug release should occur between 10 and 20 hours following injection, during which time the majority of vesicles have accumulated at the tumor and can cause toxicity [27]. In order to approximate the time constants of release of gemcitabine *in vivo*, we first explored the *in situ* release of gemcitabine from PolyGems under acidic and physiological conditions (Figure 2). A burst release of drug was observed during the first few hours, which was likely caused by the initially steep concentration gradient of gemcitabine present across the vesicle membrane, leading to a high initial diffusive flux. One explanation of this observation is that some entrapped gemcitabine in the outer PEO corona may have also contributed to the burst. Gemcitabine release was greater at all time points under acidic pH as compared to physiological pH. The PCL block is known to undergo hydrolysis of its ester linkages in solution, with a higher rate of degradation observed at acidic pHs as compared to physiological pHs [28, 29]. Consequently, both membrane permeability and the diffusive flux of gemcitabine increased at pH 5.0. The increased membrane permeability was verified with cryo-TEM images that show membrane degradation in the form of membrane pores and membrane thinning (Figure 3). Similar release curves under acidic and physiological conditions have been reported for doxorubicin release from PEO-PCL nanovesicles [14]. Understanding the release at pH 5.0 is especially important for *in vivo* delivery to tumors because the extracellular environment of many tumors is acidic [30–32]. PolyGems would likely experience both acidic and physiological pHs as they reach the tumor.

The release curves were fitted to an exponential equation of the form *c*/*c*_0_ = *α*(1 − *e* ^−*t*/*τ*^) in order to extract the time constants *τ*_pH5_ and *τ*_pH7_. This functional form is consistent with passive diffusion across a membrane. These time constants describe the time required for 63% (1 − 1/*e*) of the drug to be released due to diffusion and are functions of polymersome properties including geometric radius, membrane thickness, and diffusivity and partition coefficient of gemcitabine in the membrane. The time constants verify the quicker release observed at acidic conditions, with *τ*_pH5_ equal to 12 hours and *τ*_pH7_ equal to 16 hours. The ideal time constant for release depends on the time required for polymersomes to localize at a tumor site. Minimal drug should be released due to passive diffusion while the vesicles are still en route, and maximal and prolonged release should occur when vesicles have reached the tumor. As seen from the release curves, the majority of the gemcitabine was released within 2 days, which is consistent with the 10–20 hour window observed by Ahmed and coworkers [27].

While it is known that polymersomes will partition into tumor tissue *in vivo* [24], cellular internalization of the vesicles at the tumor is desirable so that the chemotherapeutic cargo can be released inside the cell in addition to the interstitial spaces where plasma clearance could be problematic. PZn_3_-based polymersomes have been utilized in the past to investigate polymersome uptake in dendritic cells (DCs) and DC trafficking *in vivo* [33, 34]. The uptake study of PolyGems (Figure 4) showed that vesicle internalization is a concentration- and time-dependant process with more internalization occurring for higher polymersome number and longer incubation times. However, a relatively low percentage of vesicle internalization was observed. A likely contributor is the stealth character imparted by the PEO block to the PolyGem, rendering the vesicles virtually invisible to Panc-1 cells. Another possible reason for low uptake could be a reduced cell surface area available for internalization due to cell adhesion on a tissue culture plate. Christian and coworkers observed a similar trend when incubating PZn_3_-polymersomes with dendritic cells. Negligible uptake was noticed for vesicles decorated with a PEO brush as compared to vesicles surface-conjugated with the HIV-derived TAT peptide [33]. Despite the low uptake without any targeting peptides, PolyGems performed at par with FreeGem (Figure 5). We believe that PolyGems released gemcitabine into the cell media, which was then internalized along with some vesicles via endocytosis and eventually leading to toxicity.

The true potential of PolyGems can best be determined *in vivo* where the drug kinetics are affected by all aspects of the tumor microenvironment and the therapeutic ratio (TR) can be assessed. This ratio is optimally greater than the one under *in vivo* conditions. Gemcitabine-based chemoradiotherapy in pancreatic cancer currently has a very narrow TR, being very close to one [35, 36]. The result is substantial toxicity to the GI tract. We propose that this TR can be improved in future studies using the PolyGem technology. These nano-vesicles encapsulate and release gemcitabine over a prolonged period of time, which will likely be sufficient for nonspecific accumulation of PolyGems at the tumor site due to the EPR effect. A higher local concentration of drug in the tumor interstitial fluid can be expected because the polymersome prevents significant degradation of gemcitabine while trafficking to the tumor site. We predict that endocytosis of both free drug (due to release from the vesicles) and a portion of encapsulated drug will occur. The current findings—no decreased toxicity of gemcitabine using the encapsulated agent at equal concentrations of the free agent—are encouraging and support performing *in vivo* studies. Future studies will also focus on increasing efficacy by engineered internalization (i.e., using TAT-peptides conjugated to the polymersome surface) and targeting to specific cell surface receptors.

## Figures and Tables

**Table 1:** Hydrodynamic diameter and polydispersity of different vesicle formulations.

Vesicle type	*D*_*h*_ (nm)	Polydispersity
PolyGem	180 ± 12	0.146 ± 0.045
Blank polymersome	181 ± 13	0.113 ± 0.023
PZn_3_-polymersome	180 ± 31	0.163 ± 0.020
